# P08-13 Physical activity increases social inclusion

**DOI:** 10.1093/eurpub/ckac095.126

**Published:** 2022-08-29

**Authors:** Marina Steffansson, Tuula Pehkonen-Elmi

**Affiliations:** Diaconia University of Applied Sciences, Pieksämäki, Finland

**Keywords:** physical activity, social inclusion, young people, outcomes, costs

## Abstract

**Background:**

Studies show that exercise improves self-esteem and improved self-esteem strengthens inclusion and prevents marginalization. Young people who have only completed primary school may incur additional costs of up to 370 000 euros for public administration over their lifetime, compared to those who educate themselves. In Pieksämäki, a physical activity intervention was developed for physically inactive young people not in education, employment or training (NEET) and it aims to improve their activity and thereby their social inclusion. The aim of the study was to evaluate the outcomes and the cost of the intervention.

**Methods:**

The physical activity intervention includes a personal physical exercise plan, individual instruction and free access to the swimming hall and gym during the participation. Each participant gets as much guidance as they need. We present results only for three cases, because the target group was very challenging. The follow-up period was 12 months.

**Results:**

To evaluate the outcomes we used three subjective measures, 3X10D® (survey-self-evaluation tool), the Abilitator®, and 15D (HRQoL, the health-related quality of life instrument). The 3X10D® showed that life as a whole and self-esteem improved in two cases. In all three cases managing in daily activities improved. However, the Abilitator® showed slight improvement in wellbeing and clear improvement in physical functioning in all three cases. We obtained 15D results only from two cases. In one case HRQoL decreased slightly and in the other one it increased significantly. The employment status of these three cases changed. In the beginning there was an unemployed person, a person having only 9-year basic education and a drop-out from vocational education. After the intervention the unemployed person was working, and the other two were studying. Depending on the number of visits to the physical exercise instructor and other physical activities the costs of the intervention varied between 1042 and 1215 euros.

**Conclusions:**

Based on the results physical functioning and self-esteem improved and that can lead to stronger social inclusion. Although the three individuals underwent intensive individual instruction, the costs were low compared to the costs of social exclusion reported in earlier researches.

